# Characterization of a Novel Small Molecule Subtype Specific Estrogen-Related Receptor α Antagonist in MCF-7 Breast Cancer Cells

**DOI:** 10.1371/journal.pone.0005624

**Published:** 2009-05-20

**Authors:** Michael J. Chisamore, Michael E. Cunningham, Osvaldo Flores, Hilary A. Wilkinson, J. Don Chen

**Affiliations:** 1 Department of Molecular Endocrinology, Merck Research Laboratories, West Point, Pennsylvania, United States of America; 2 Department of Integrative Systems of Neuroscience, Merck Research Laboratories, West Point, Pennsylvania, United States of America; 3 Department of Pharmacology, University of Medicine and Dentistry of New Jersey, Piscataway, New Jersey, United States of America; Bauer Research Foundation, United States of America

## Abstract

**Background:**

The orphan nuclear receptor estrogen-related receptor α (ERRα) is a member of the nuclear receptor superfamily. It was identified through a search for genes encoding proteins related to estrogen receptor α (ERα). An endogenous ligand has not been found. Novel ERRα antagonists that are highly specific for binding to the ligand binding domain (LBD) of ERRα have been recently reported. Research suggests that ERRα may be a novel drug target to treat breast cancer and/or metabolic disorders and this has led to an effort to characterize the mechanisms of action of N-[(2Z)-3-(4,5-dihydro-1,3-thiazol-2-yl)-1,3-thiazolidin-2-yl idene]-5H dibenzo[a,d][7]annulen-5-amine, a novel ERRα specific antagonist.

**Methodology/Principal Findings:**

We demonstrate this ERRα ligand inhibits ERRα transcriptional activity in MCF-7 cells by luciferase assay but does not affect mRNA levels measured by real-time RT-PCR. Also, ERα (*ESR1*) mRNA levels were not affected upon treatment with the ERRα antagonist, but other ERRα (*ESRRA*) target genes such as pS2 (*TFF1*), osteopontin (*SPP1*), and aromatase (*CYP19A1*) mRNA levels decreased. *In vitro*, the ERRα antagonist prevents the constitutive interaction between ERRα and nuclear receptor coactivators. Furthermore, we use Western blots to demonstrate ERRα protein degradation via the ubiquitin proteasome pathway is increased by the ERRα-subtype specific antagonist. We demonstrate by chromatin immunoprecipitation (ChIP) that the interaction between *ACADM*, *ESRRA*, and *TFF1* endogenous gene promoters and ERRα protein is decreased when cells are treated with the ligand. Knocking-down ERRα (shRNA) led to similar genomic effects seen when MCF-7 cells were treated with our ERRα antagonist.

**Conclusions/Significance:**

We report the mechanism of action of a novel ERRα specific antagonist that inhibits transcriptional activity of ERRα, disrupts the constitutive interaction between ERRα and nuclear coactivators, and induces proteasome-dependent ERRα protein degradation. Additionally, we confirmed that knocking-down ERRα lead to similar genomic effects demonstrated *in vitro* when treated with the ERRα specific antagonist.

## Introduction

ERRα is an orphan member of the superfamily of hormone nuclear receptors. The ERR subfamily consists of three members, ERRα, ERRβ, and ERRγ. ERRα was one of the first orphan receptors identified. It was found by using the DNA-binding domain (DBD) of Estrogen Receptor α (ERα) as a hybridization probe to screen recombinant DNA libraries [1]. Amino acid sequence comparison shows that apart from ERRβ and ERRγ, ERRα is more closely related to ERα and ERβ than any other member of the superfamily of nuclear hormone receptors. ERRα and both ERα and ERβ DNA Binding Domains share 70% amino acid identity. ERRα and ERα Ligand Binding Domains (LBD) share 36% amino acid identity; while ERRα and ERβ LBD's share 37% amino acid identity [2], [3]. In addition, although ERs and ERRs share a number of similar biochemical properties, ERRs do not bind 17β-estradiol (E2).

ERRα is known to bind to DNA as either a monomer or a dimer. ERRα can bind to estrogen-response elements (ERE) containing the recognition motif AGGTCAnnnTGACCT; ERRα also recognizes the single consensus half-site sequence TNAAGGTCA, referred to as an ERR-response element (ERRE) [4]. ERRα can bind the inverted repeat ERE as a dimer [5]. The binding of ERRα to an ERE or ERRE can lead to either a stimulatory or repressive event depending on the cell type, response element, context within a specific promoter, phosphorylation state of the receptor, potential ligands present, genomic context of ERRα (either competing or cooperating with ERα for binding), other receptors and coregulators present, and additional transcription factors involved [2]. Consequently, ERRs and ERs share common target genes (such as pS2, lactoferrin, and osteopontin) and exhibit cross-talk [6], [7], [8], [9].

Whereas many other members of the steroid receptor superfamily are activated by ligand (including ERs), ERRs are constitutively active without the addition of a specific ligand. ERRα and ERRβ have been shown to be constitutive activators of the classic ERE [10]. The authors also demonstrate that the p160 cofactors AIB1 (also known as SRC-3, NCoA3, ACTR, RAC3), GRIP1 (also known as SRC-2, NCoA2, TIF2) and SRC-1 (also known as NCoA1) potentiate the transcriptional activity by ERRα. It has been reported [9], [10] using glutathione S-transferase (GST) pull down assays that ACTR (AIB1), SRC-1, and GRIP1 interact with the AF-2 domain of the LBD of ERRα without the addition of exogenous ligand. Moreover, fluorescence resonance energy transfer (FRET) assay has been used to demonstrate that SRC-1 and SRC-2 (GRIP-1) interact with all three ERRs without the addition of exogenous ligand. While ligands are not required for activation of ERR activity, there are known ligands which can modulate ERRs. Diethylstilbestrol (DES) antagonizes all three ERR isoforms whereas 4-hydroxytamoxifen (4-OHT) is an isoform specific inhibitor of ERRβ and ERRγ [11], [12], [13].

In addition to the p160 family of nuclear receptor coactivators that modulate ERR activity, another class of coactivators has also been reported. This class is made up of Proliferator-activated Receptor γ Coactivator-1 α (PGC-1α) [14], [15], [16], [17] and Proliferator-activated Receptor γ Coactivator-1 β (PGC-1β) [18]. PGC-1α and PGC-1β are important regulators of genes that control many key aspects of metabolism including glucose uptake, gluconeogenesis, mitochondrial biogenesis, adipocyte cell fate specification, and adaptive thermogenesis [19]. PGC-1α interacts with ERRα and potentiates its transcriptional activity [14], [15], [16], [17]. In a direct comparison of the binding affinities of SRC-1 and PGC-1α to bind ERRα, it has been shown that ERRα binds PGC-1α with 140-fold increased affinity in comparison to SRC-1 [20].

We have previously reported ERRα-subtype selective antagonist ligands [21]. Other known ERRα ligands include the synthetic estrogen DES [13] and the ERRα selective inverse agonist, XCT790 [22]. It has been established that XCT790 induces ubiquitin proteasome dependent ERRα degradation [23]. An additional inverse agonist, Cyclohexylmethyl-(1-p-tolyl-1H-indol-3-ylmethyl)-amine, has been co-crystallized with the human ERRα LBD and the authors describe a novel molecular mechanism of action for inverse agonism of ERRα [24]. Moreover, it is known that the selective estrogen receptor modulator (SERM) 4-OHT is an antagonist to ERRβ and ERRγ in FRET and cell-based reporter assays [11], [12]. Over the past several years the scientific literature has continued to suggest that the orphan nuclear receptor ERRα could represent an important target for the treatment of breast cancer [2], [3], [25]. Novel ERRα-subtype specific antagonists that are highly specific for the ligand binding domain (LBD) of ERRα have been recently reported [21]. The possible important use for these ligands has led to an effort to study mechanisms of action of ERRα antagonists. In particular, N-[(2Z)-3-(4,5-dihydro-1,3-thiazol-2-yl)-1,3-thiazolidin-2-yl idene]-5H dibenzo[a,d][7]annulen-5-amine, which has a strong antagonistic effect on the constitutive interaction between ERRα and nuclear coactivators was identified [21]. For simplicity, in the present study N-[(2Z)-3-(4,5-dihydro-1,3-thiazol-2-yl)-1,3-thiazolidin-2-yl idene]-5H dibenzo[a,d][7]annulen-5-amine will be called “Compound A”.

Our studies demonstrate that Compound A antagonizes ERRα transcriptional activity but shows little affect on ERRα mRNA levels. ERα mRNA and protein levels were not affected upon treatment with the ERRα antagonist, but other ERRα target genes such as pS2, osteopontin, and aromatase mRNA levels decreased upon treatment with the ERRα-subtype specific ligand. In addition, this ERRα tri-cyclic ligand antagonizes the constitutive interaction between ERRα and nuclear coactivators. We provide evidence that ERRα protein degradation is induced by the ERRα-subtype specific antagonist and this degradation is mediated though the ubiquitin 26S proteasome pathway. We report that the interactions between ERRα protein and the endogenous ERRα responsive gene promoters (*ESRRA*, *ACADM* and *TFF1*) are decreased by treatment with Compound A. Lastly, knocking-down ERRα by shRNA led to similar genomic effects seen when MCF-7 cells were treated with our ERRα antagonist.

## Materials and Methods

### Cell Lines and Reagents

The MCF-7 cell line (obtained from ATCC, Manassas, VA), and MCF-7/shRNA ERRα RNAi cell lines were maintained in EMEM with Earle's BSS and 2 mM L-glutamine that was modified to contain 1.0 mM sodium pyruvate, 0.1 mM nonessential amino acids, and 1.5 g/L sodium bicarbonate. It was also supplemented with 10% fetal bovine serum, 10 ug/ml bovine insulin, 100 units/ml of penicillin/streptomycin, at 37°C and 5% CO_2_. All cell lines were determined to be free of mycoplasma. Experiments that required maintenance of cells in “stripped media,” were washed with phosphate buffered saline, and then the media was changed to EMEM without phenol red that contained 10% charcoal dextran – treated FBS (CD-FBS).

### Transient Transfection and Luciferase Assay

MCF-7 cells were maintained in phenol free EMEM/CD-FBS media for 4 days prior to performing transfections. 25,000 cells per well were plated into 96-well plates. 0.125 ug pGL2 Luc (Promega), or 0.125 ug p3xERE-TK-LUC [pGL2 plasmid (Promega, Madison, WI) containing 3 tandem repeats of the estrogen response element (ERE) sequence 5′ – TTTGATCAGGTCACTGTGACCTCTAGAGT-3′, placed upstream of a minimal herpes simplex thymidine kinase (TK) promoter directing the expression of the luciferase coding sequence (a generous gift from Tina Chang, Merck Research Laboratories, Rahway, NJ) and 0.0125 ug of phRL-TK renilla plasmid (Promega) were co-transfected in triplicate wells along with (when indicated) either 0.0625 ug pcDNA 3.1 (Invitrogen, Carlsbad, CA) or 0.0625 ug pcDNA3.1 hERRα (described below). Plasmids were diluted in OptiMEM (Invitrogen), the transfection reagent Lipofectamine LTX (Invitrogen) was added to the DNA solution and incubated for 25 minutes at room temperature. Next, the DNA-Lipofectamine complexes were added to the cells and incubated overnight. Subsequently, the cells (in triplicate) were treated with vehicle (DMSO), 100 pM 17-beta-estradiol (Sigma), 5 uM Compound A (Merck & Co, West Point, PA), 100 pM 17-beta-estradiol/5 uM Compound A, or 1 uM ICI-182,780 (faslodex) (Tocris, Ellisville, MO) for 48 hours. Cells were then harvested and cell lysates were assayed for luciferase activity (renilla normalized) as per the manufacturer's directions by utilizing the Dual-Luciferase Reporter Assay System (Promega) and the Wallac Victor plate reader (Perkin Elmer, Wellesley, MA).

### Real-time RT-PCR

MCF-7 cells were maintained in phenol free EMEM/CD-FBS media for 4 days prior to drug treatments (in triplicate) and MCF-7/shRNA ERRα RNAi cells were maintained in normal media containing whole serum (described above). Total RNA was extracted from MCF-7 cells treated with either DMSO (Control) or 5 uM Compound A for 24 or 48 hours. The RNA samples were DNase I (Ambion Inc., Austin, TX) treated and cDNA was synthesized (High-Capacity cDNA Archive Kit, Applied Biosystems). Real-time RT-PCR was performed with an ABI 7900 HT sequence detection system (Applied Biosystems, Foster City, CA). Primer/probe sets for target genes: human ERRα (*ESRRA*) (Hs00607062_gH), human ERα (*ESR1*) (Hs00174860_m1), human PGC-1α (*PPARGC1A*) (Hs00173304_m1), human PDK4 (*PDK4*) (Hs00176875_m1), human osteopontin (*SPP1*) (Hs00959010_m1), human pS2 (*TFF1*) (Hs00170216_m1), human ACADM (*ACADM*) (Hs00163494_m1) and 18S rRNA endogenous control (4308329) were purchased from Applied Biosystems. The housekeeping gene18S rRNA was used as the internal quantitative control for normalization. Relative gene expression was calculated with the ΔΔCt method as outlined in the Applied Biosystems User Guide. In brief, the threshold cycle (C_T_) values for the target gene and reference (18S) were determined by ABI PRISM Sequence Detection System software. Mean C_T_ values and standard deviations were calculated in Microsoft Excel. ΔC_T_ was calculated by ΔC_T_ = C_T_ target−C_T_ reference. After the mean and standard deviation of the ΔC_T_'s value were determined, ΔΔC_T_ = ΔC_T_ test sample−ΔC_T_ calibrator sample. Next, the standard deviations of the ΔΔC_T_ values were calculated and finally, the fold-differences were determined by the ΔΔC_T_, expressed as 2^−ΔΔCT^.

### Expression, Purification, and Biotinylation of Fusion Proteins

Bacterial expression plasmid constructs GST-AIB1 RID, GST-GRIP-1 RID, GST-PGC-1α RID along with bacterial growth, fusion protein expression, purification, cleavage of GST, and biotinylation of AIB1, GRIP-1, and PGC-1α have been previously described [21].

### ERRα and ERα Expression Plasmids

For construction of the pcDNA3.1 hERRα expression plasmid, full-length human ERRα was amplified from human brain Marathon-ready cDNA (Clonetech Laboratories Inc.) by using forward primer 5′-GGGAAGCTTAGGTGACCAGCGCCATGTCCAGCCAGG-3′, reverse primer 5′- GGGGAATTCACCCCTTGCCTCAGTCCATCATGGCCTCG-3′, and cloned into mammalian expression vector pcDNA3.1(+) (Invitrogen) (Generous gift from Dr. Sheng-Jian Cai, Merck Research Laboratories, Rahway, NJ). For the pcDNA3.1 hERα expression plasmid, the full-length human ERα was amplified from human liver cDNA (amino acids 1–595) and initially cloned into the BamH1 and SpeI sites in the phagemid vector pBlueScript KS (-) (Stratagene, La Jolla, CA). Subsequently, full-length human ERα (a.a. 1–595) was subcloned as an EcoRV – SpeI fragment into EcoRV – XbaI sites of the mammalian expression vector pcDNA3.1(+) (Invitrogen, Carlsbad, CA).

### ERRα Antibody

The ERRα specific peptide sequence: AGPLAVAGGPRKTAAPVN, was synthesized by EvoQuest Custom Antibody Services (Invitrogen). The peptide was then coupled to a hapten carrier (keyhole limpet hemocyanin) for immunization. Polyclonal antibodies were generated in New Zealand white rabbits and the polyclonal ERRα specific antibody (pAb ERRα) was purified by affinity chromatography.

### *In Vitro* Expression of ERα and ERRα

Full-length human ERα (pcDNA3.1 hERα) and full-length human ERRα (pcDNA3.1 hERRα) proteins were expressed by a TnT coupled reticulocyte lysate system as per the manufacturer's recommended conditions (Promega Corporation) for use as positive controls with Western Blotting. In addition, [^35^S]methionine was added to the transcription/translation reaction for radiolabeled hERRα that was used in the biotinylated pull-down assays.

### Biotinylated Pull-down Assay

ProFound Pull-Down Biotinylated Protein:Protein Interaction Kit (Pierce, Rockford, IL) was utilized and manufacture's instructions were followed. Briefly, streptavidin beaded agarose (washed with 1× TBS) was incubated with biotinylated protein in 1× TBS at 4°C in provided spin column for 1 hour on a rocking platform and then columns were centrifuged. Biotin blocking solution was added, samples were incubated and centrifuged. Biotin blocking step was repeated once followed by washes with 1× TBS. *In vitro* translated [^35^S]-labeled protein in 1× TBS was added along with DMSO (control), 10 µM Compound A, or 10 µM DES to biotinylated protein bound to streptavidin beaded agarose. The samples were then incubated for 4 hours at 4°C on a rocking platform. The bound protein was washed with 1× TBS and the beads were collected by centrifugation. The bound protein was eluted in SDS sample buffer, loaded into a 10% NuPage Bis-Tris Gel (Invitrogen) and analyzed by phosphorimaging (Typhoon 9400, ImageQuant TL software, GE Healthcare). Positive control, 10% input, *in vitro* translated full-length human ERRα [^35^S]-labeled protein. Negative control, sulfo-NHS-LC-biotin pulled down with streptavidin beaded agarose and incubated with ERRα [^35^S]-labeled protein. Equal loading was determined by staining 10% NuPage Bis-Tris Gels with Coomassie brilliant blue stain.

### Chromatin Immunoprecipitation (ChIP) Assay

After 4 days of growing the MCF-7 cells in EMEM without phenol red that contained 10% CD-FBS, the cells were treated with DMSO or 5 uM Compound A in duplicate for 24 and 48 hours. Subsequently, the cells were fixed according to Genpathway, Inc. cell fixation protocol which can be found at www.genpathway.com and the chromatin immunoprecipitation were carried as described (43) except ERRα antibody (described above) or GRIP-1 (Santa Cruz Biotechnology, M-343) were used for the immunoprecipitation. Primers used for quantitative real-time PCR (Q-PCR) were as follows: ERRα forward, 5′ – CTT CCC CGT GAC CTT CAT T – 3′, ERRα reverse, 5′ – AGC CGA CTT AAA ACA TGC AAT A – 3′; ACADM forward, 5′ – AAC GCA GAA AAC CAA ACC AG – 3′, ACADM reverse, 5′ – CAT GCT CCG TGA CCC TTG; pS2 forward, 5′ – ACA TGG AAG GAT TTG CTG ATA – 3′, pS2 reverse, 5′ – TTC CGG CCA TCT CTC ACT AT – 3′, and Untr12 forward, 5′ – TGG ACC TTT ACC TGC TTT ATC A – 3′ and reverse, 5′ – AGC AAG GAC TAG GAT GAC AGA A – 3′. All Q-PCR amplifications were performed in triplicate.

### Analysis of ERα and ERRα Protein Levels

MCF-7 cells were grown in phenol free EMEM/CD-FBS media for 4 days prior to drug treatments and MCF-7/shRNA ERRα RNAi cells were maintained in normal media containing whole serum (described above). MCF-7 cells were treated with vehicle (DMSO), 10 nM 17-beta-Estradiol (Sigma), 10 nM 4-hydroxytamoxifen (Sigma), 10 nM ICI-182,780 (Tocris), or 5 uM Compound A (Merck & Co., West Point, PA) for 12, 24, or 48 hours. Nuclear protein extractions were carried out according to the protocol of the NE-PER Nuclear Extraction Kit (Pierce). For the protein degradation assay MCF-7 cells were treated with vehicle (DMSO), 5 uM Compound A, 1 uM MG132 (Sigma), or 5 uM Compound A/1 uM MG132 for 36 hours. RIPA buffer (Pierce) was utilized to obtain whole cell extractions by following the manufacturer's protocol. Protein concentration was determined with the DC Protein Assay (Bio-Rad, Hercules, CA) and 20 ug of nuclear protein extracts or 40 ug of whole cell protein extracts were loaded into a 10% NuPage Bis-Tris Gel (Invitrogen). After electrophoresis, the proteins were transferred to nitrocellulose membrane (Invitrogen). Western blotting was carried out by utilizing pAb ERRα (described above) or ERα (G-20, Santa Cruz Biotechnology, Santa Cruz, CA), ECL rabbit IgG, HRP-linked whole antibody (from donkey) (GE Healthcare, Piscataway, NJ), and Western Lightning Chemiluminescence Reagent Plus (Perkin Elmer, Boston, MA). Equal loading of nuclear protein extracts per lane was assessed by Coomassie Brilliant Blue staining of a gel run in parallel while equal loading of whole cell protein extracts per lane was assessed by stripping the nitrocellulose membrane and re-probing with β-actin monoclonal antibody (Sigma). Densitometric quantification of protein levels from three independent experiments were performed by using the AlphaEase FC Imaging Software program (Alpha Innotech, San Leandro, CA). MCF-7/shRNA ERRα RNAi cell line nuclear protein extraction, protein concentration determination, electrophoresis, protein transfer, and Western blotting are described above.

### Stable Transfection with shRNA ERRα and shRNA (-) Plasmids

0.5 ug of four unique SureSilencing shRNA plasmids (SuperArray Bioscience Corporation, Frederick, MD) specific for human ERRα and a negative control were transfected separately into 40,000 MCF-7 cells per well (24-well plate). shRNA (-) and shRNA ERRα plasmids were under the control of the U1 promoter and also contain GFP. Plasmids were diluted in OptiMEM (Invitrogen), the transfection reagent Lipofectamine LTX (Invitrogen) was added to the DNA solution and incubated for 25 minutes at room temperature. Next, the DNA-Lipofectamine complexes were added to the cells, incubated for 24 hours, and then cells were replenished with fresh media. Subsequently, transfected cells were expanded.

### FACS Sorting of MCF-7/shRNA ERRα RNAi Cells

Cells were resuspended in DPBS (Gibco 14190), 0.1% BSA, 25 mM HEPES; GFP-enriched cells were sorted on a FACSVantage-SE flow cytometer (Becton Dickinson, San Jose, CA) equipped with an Innova 70C-4 (488 nm) argon ion laser (Coherent, Palo Alto, CA). GFP was excited at 488 nm and fluorescence emission was detected using a 530/30 BP filter. Data from the experiments were analyzed with CellQuest software (Becton Dickinson). GFP expressing cells underwent 4 rounds of GFP enrichment by FACS. Extent of ERRα knock-down was measured in triplicate by real-time RT-PCR (described above).

### Statistics

Error bars represent standard error of the mean (SEM) between replicates of a given experiment. Comparisons between two groups were made by analysis of variance (ANOVA) followed by a student t-test at 0.05 significance level with P values indicated.

## Results

### Compound A Inhibits Constitutive Transcriptional Activity of ERRα

**Figure 1:**
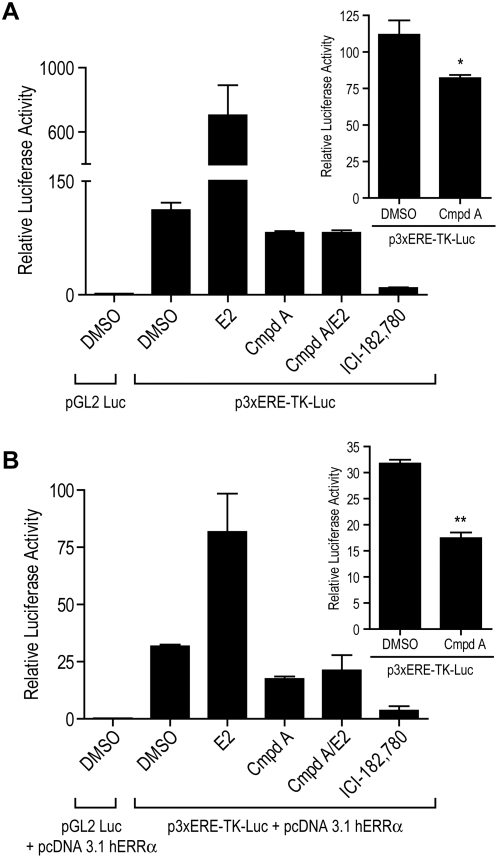
ERRα antagonist Compound A inhibits constitutive transcriptional activity of ERRα. (A) Plasmids pGL2 Luc ERE (empty vector control) or p3xERE-TK-LUC and phRL-TK renilla plasmid were co-transfected into MCF-7 cells. After indicated cell treatment, cells were harvested and cell lysates were assayed for luciferase activity (renilla normalized). Cells transfected with the p3xERE-TK-Luc showed constitutive activity compared to the empty vector pGL2 Luc treated with vehicle. MCF-7 cells transfected with the p3xERE-TK-Luc and treated with E2 conferred a 6.3-fold increase in transcriptional activity while cells treated with Compound A are significantly repressed, 0.73-fold (or 27% decrease) below basal level (*, P = 0.032) (Fig. 1A inset) or when treated with Compound A in combination with E2, Compound A still represses the transcriptional effect conferred by ERRα. ICI - 182,780 also greatly reduces any transactivation. (B) The same experiment (described above) was performed along with either pcDNA 3.1 (empty vector control) or pcDNA3.1 hERRα as indicated. Over-expressing ERRα in MCF-7 cells increases down modulation (relative luciferase activity) within all treatment groups. Cells transfected with the p3xERE-TK-Luc+pcDNA 3.1 hERRα conferred an 114-fold increase in transcriptional activity above cells transfected with pGL2 Luc and treated with vehicle. Cells transfected with the p3xERE-TK-Luc+pcDNA 3.1 hERRα and treated with E2 exhibited a 2.6-fold increase in transcriptional activity in comparison to pGL2 Luc+pcDNA3.1 hERRα. Over expressing ERRα leads to a 45% decrease in transactivation upon treatment with Compound A (P = 0.001) (Fig. 1B inset). Similarly, Cmpd A+E2 led to a 33% decrease and over expressing ERRα in MCF-7 cells still led to nearly abolishing transactivation upon treatment with ICI 182,780. Results are expressed as the normalized luciferase activity (mean±SEM) of three independent experiments performed in triplicate. Differences in luciferase activity between vehicle (DMSO) and Cmpd A were measured by ANOVA followed by a student t-test with a 0.05 significance level. *, P = 0.032 and **, P = 0.001.

ERRα binding to ERE's and subsequent constitutive transactivation has been shown [10], [13], [26]. In addition, Kraus et al [26] has reported that ERRα can modulate estrogen responsiveness and effectively compete with ERα for the binding to EREs. To test whether our novel ERRα ligand antagonizes the constitutive transcriptional activity of ERRα, we cotransfected MCF-7 breast cancer cells with the reporter plasmid p3xERE-TK-Luc or the ERE-negative control plasmid (pGL2 Luc) together with the control parental vector pcDNA3.1 and phRL-TK renilla plasmid for normalization. The co-transfected cells were then treated with vehicle (DMSO), 100 pM E2, 5 uM Compound A, 100 pM E2/5 uM Compound A, or 1 uM ICI - 182,780. After 48 hours cells were harvested and cell lysates were assayed for luciferase activity. Cells transfected with the p3xERE-TK-Luc and treated with DMSO demonstrated constitutive (basal level) activity with a 73-fold increase in transcriptional activity above cells transfected with pGL2 Luc and treated with DMSO (Fig. 1A). MCF-7 cells transfected with the p3xERE-TK-Luc and treated with E2 had a 6.3-fold increase in transcriptional activity verses the basal level. Cells treated with the ERRα antagonist, Compound A are transcriptionally repressed, 0.73-fold (or 27% decrease) below basal level (*P* = 0.032) (Fig. 1A inset).

To study the effect of the ERRα-subtype specific antagonist on estrogen dependent transcriptional activity, cells were treated with E2 in combination with the ERRα antagonist (Fig. 1A). When cells were treated with estrogen plus Compound A there was a reduction in transcriptional activation. Additionally, the estrogen receptor selective antagonist, ICI - 182,780, greatly reduces any transactivation suggesting that ER is needed for estrogen stimulated expression to occur at the ERE (Fig. 1A). Thus, taken together, the functionality of the interconnections/cross-talk of ERRα and ERα at an ERE is illustrated.

Would having more ERRα present lead to greater antagonism by Compound A? To answer this question, a similar co-transfection experiment described above was performed and the ERRα expression plasmid (pcDNA3.1 hERRα) was co-transfected (instead of control parental vector pcDNA3.1) with either p3xERE-TK-Luc or pGL2 Luc. Over-expressing ERRα in MCF-7 cells leads to decreased down modulation within all treatment groups relative to luciferase activity (compare Fig. 1A to 1B). Cells transfected with the p3xERE-TK-Luc+pcDNA 3.1 hERRα demonstrated constitutive (basal level) activity by conferring an 114-fold increase in transcriptional activity above cells transfected with pGL2 Luc and treated with vehicle (Fig. 1B). MCF-7 cells transfected with the p3xERE-TK-Luc+pcDNA 3.1 hERRα and treated with 100 pM E2 conferred a 2.6-fold increase in transcriptional activity verses the basal level. Moreover, while treating cells with 5 uM Compound A lead to a 27% decrease below the basal constitutive activity in the original experiment (Fig. 1A); over expressing ERRα leads to a 45% decrease in transactivation upon treatment with 5 uM Compound A (*P* = 0.001) (Fig. 1B inset). Similarly, 5 uM Compound A plus 100 pM E2 also led to a 33% decrease. Therefore, while transfecting ERRα into MCF-7 cells leads to an overall decreased modulation of estrogen responsiveness (also previously reported [26]), a larger window of antagonism was also demonstrated with more ERRα present. Over expressing ERRα in MCF-7 cells nearly abolishes transactivation upon treatment with 1 uM ICI 182,780 (Fig. 1B).

### Compound A Suppresses Expression of ERRα Target Genes

**Figure 2:**
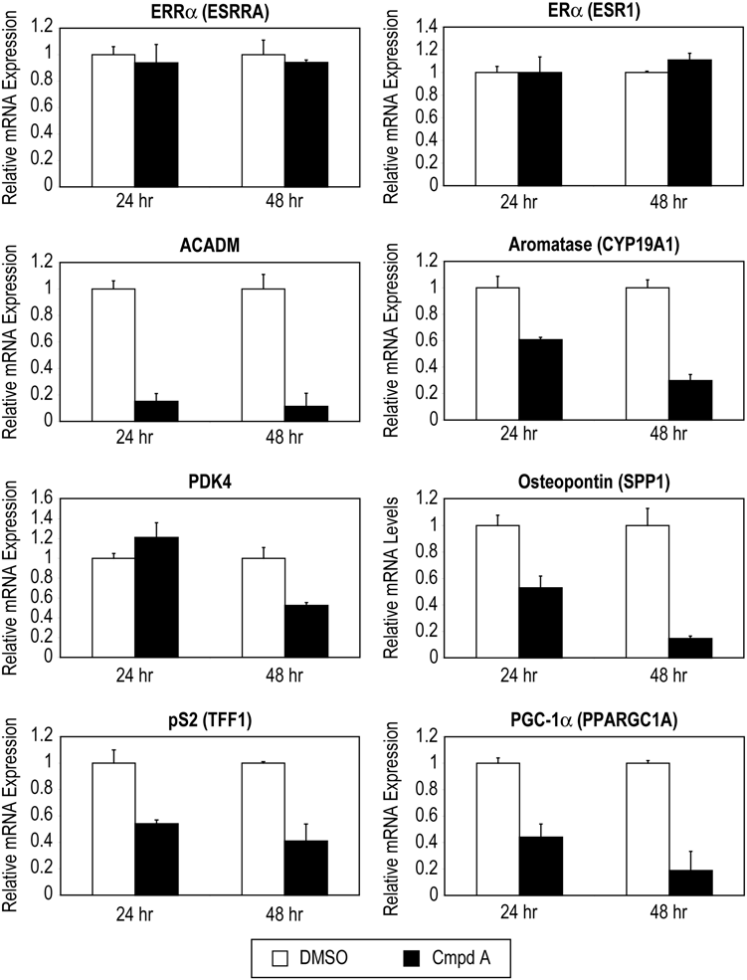
Compound A treatment effects on mRNA levels of ERRα target genes. MCF-7 breast cancer cells were treated with Compound A for either 24 or 48 hours. No change in ERRα or ERα mRNA levels were measured after 24 and 48 hours of treatment with the ERRα antagonist verses vehicle (DMSO), while other ERRα target genes medium-chain acyl coenzyme (*ACADM*), aromatase (*CYP19A1*), pyruvate dehydrogenase kinase 4 (*PDK4*), osteopontin (*SPP1*), and pS2 (*TFF1*) were all significantly (P<0.001) down modulated upon treatment with ERRα antagonist at 24 and/or 48 hours. Additionally, peroxisome proliferator-activated receptor coactivator-1α (PGC-1α) (*PPARGC1A*), is also significantly (P<0.001) down modulated upon treatment with Compound A. These results are representative of three independent experiments performed in triplicate. Differences in relative mRNA expression between vehicle (DMSO) and Cmpd A were measured by ANOVA followed by a student t-test with a 0.05 significance level.

Since Compound A was shown to inhibit the constitutive transcriptional activity of ERRα (Fig. 1), we next wanted to examine the effects of Compound A on target gene expression at the mRNA level. Quantitative real-time RT-PCR was used to measure ERRα (*ESRRA*) target gene mRNA levels in MCF-7 cell treated with 5 uM Compound A for either 24 or 48 hours. No reduction in steady-state ERRα or ERα (*ESR1*) mRNA levels was measured after 24 and 48 hours of treatment with the ligand (Fig. 2, S1). On the contrary, other known ERRα target genes including medium-chain acyl coenzyme (*ACADM*) [4], aromatase (*CYP19A1*) [27], pyruvate dehydrogenase kinase 4 (*PDK4*) [28], osteopontin (*SPP1*) [7], and pS2 (*TFF1*) [6] were all down modulated upon treatment with the compound for 48 hours (Fig. 2, S1). Additionally, peroxisome proliferator-activated receptor coactivator-1α (PGC-1α) (*PPARGC1A*), although not considered an ERRα target gene, is known to bind and interact with ERRα [14], [29], was robustly down modulated upon treatment with the ERRα antagonist in comparison to cells treated with vehicle alone (Fig. 2, S1).

### Compound A Decreased Constitutive Interactions of ERRα with Nuclear Coactivators AIB1, GRIP-1 and PGC-1α

**Figure 3:**
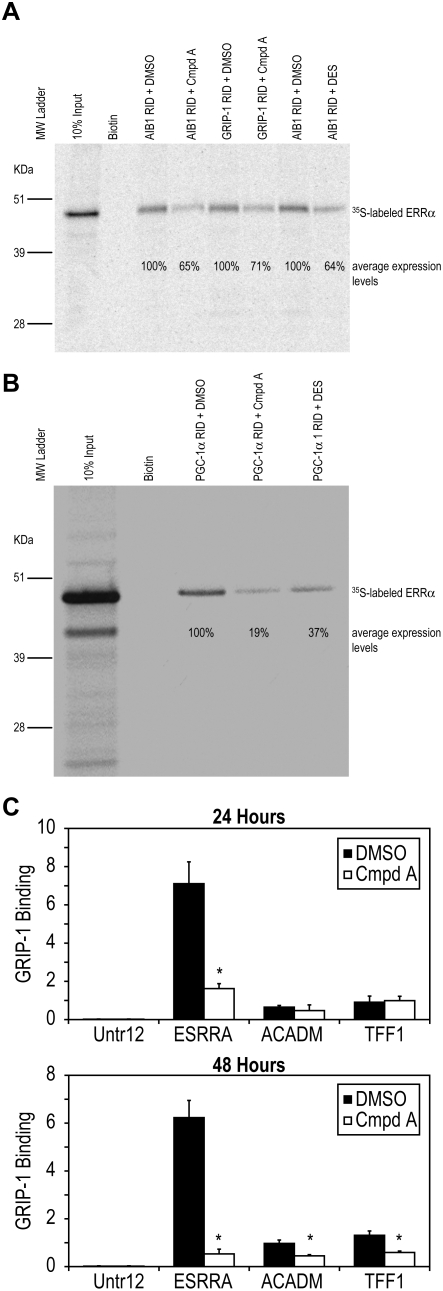
Constitutive interaction of ERRα and nuclear coactivators is reduced upon treatment with ERRα antagonist. Biotinylated pull-down assays with nuclear coactivators AIB1, GRIP-1 (Fig. 3A), and PGC-1α (Fig. 3B). Lane 1 molecular weight ladder. Lane 2, 10% input, *in vitro* translated full-length human ERRα ^35^S labeled protein. Lane 3, sulfo-NHS-LC-biotin plus ^35^S-labeled ERRα. (A) Treatment with Compound A; AIB1 RID/ERRα interaction showed a 35% reduction in comparison to the vehicle (DMSO) treated sample (lane 4 and 5), GRIP-1 RID/ERRα interaction was reduced by 29% (lane 6 and 7). Similarly, AIB1 RID/ERRα interaction showed a 36% reduction in comparison to the vehicle (DMSO) when treated with DES (lane 8 and 9). (B) Treatment with Compound A; PGC-1α RID/ERRα interaction exhibited a 81% reduction in comparison to the vehicle (DMSO) treated sample (lane 4 and 5). Additionally, PGC-1α RID/ERRα interaction displayed a 37% reduction in comparison to the vehicle (DMSO) when treated with DES (lane 4 and 6). Pull-down assays represent three independent experiments yielding similar results. (C) MCF-7 cells treated with vehicle (DMSO) or Compound A for 24 and 48 hours. Chromatin was immunoprecipitated with anti-GRIP-1 antibody, and quantitative real-time PCR (Q-PCR) was performed with primers targeting estrogen-related receptor response elements (ERREs) in ERRα (*ESRRA*), *ACADM*, and pS2 (*TFF1*). At 24 hours, treatment with Compound A significantly decreased binding of GRIP-1/ERRα (*ESRRA*) (DMSO vs. Cmpd A, P<0.001), while at 48 hours significantly decreased binding of ERRα (*ESRRA*), ACADM (*ACADM*), and pS2 (*TFF1*)/GRIP-1 (DMSO vs. Cmpd A, P<0.001) was exhibited. All ChIP experiments were independently repeated yielding reproducible results. Differences in binding between vehicle (DMSO) and Cmpd A were measured by ANOVA followed by a student t-test with a 0.05 significance level.

Our group has previously reported an IC_50_ of 170 nM for Compound A in an ERRα LBD/PGC-1α coactivator homogenous time-resolved fluorescence interaction assay [21]. To further study this inhibitory effect we performed biotinylated pull-down assays with AIB1, GRIP-1, and PGC-1α nuclear coactivators to look at the effects of Compound A on ERRα/nuclear coactivator constitutive interaction. The receptor interaction domains (RID) of AIB1 (aa 557–773), GRIP-1 (aa 565–798), and PGC-1α (aa 1–338) were expressed, biotinylated, and purified. Full-length human ERRα protein was expressed and ^35^S[methionine] labeled. After incubating ERRα, nuclear coactivator, and 10 uM ligand, a standard streptavidin bead/biotinylated pull-down assay was carried out (see Materials and Methods). The constitutive interaction of ERRα with nuclear coactivators AIB1, GRIP-1, or PGC-1α was unaffected by the presence of DMSO, but was considerably reduced upon treatment with Compound A or the known ERRα antagonist DES [11], [13] (Fig. 3). Upon treatment with Compound A, AIB1 showed a 35% reduction in comparison to the vehicle (DMSO) treated sample (Fig. 3A). Similar results were also seen with nuclear coactivators GRIP-1 (Fig. 3A) or PGC-1α with either Compound A or DES (Fig. 3B). The nuclear coactivator PGC-1α exhibited the greatest release with an 81% reduction (Fig. 3B). The reduction of nuclear coactivator levels upon treatment with the ERRα antagonist demonstrates disruption of the constitutive interaction between ERRα and these coactivators.

To further support the finding that Compound A disrupts the constitutive interaction between ERRα and nuclear coactivators, we also performed ChIP assays after MCF-7 cells were treated with vehicle (DMSO) or 5 uM Compound A for 24 and 48 hours. The cells were fixed, chromatin was immunoprecipitated with anti-GRIP-1 antibody, and quantitative real-time PCR (Q-PCR) was performed with primers targeting well-studied estrogen-related receptor response elements (ERREs) in ERRα (*ESRRA*) [30], [31], ACADM (*ACADM*) [4], [32], and pS2 (*TFF1*) promoters [6], [33]. At 24 hours, treatment with Compound A significantly decreased association of ERRα/GRIP-1 (P<0.001) (Fig. 3C top) to a region in ESSRA (ERRα gene), while at 48 hours decreased association events of ERRα/GRIP-1 to genomic regions flanking *ESSRA*, *ACADM*, and *TFF1* (DMSO vs. Cmpd A, *P*<0.001) (Fig. 3C bottom) were detected.

### Compound A Induces ERRα Protein Degradation

**Figure 4:**
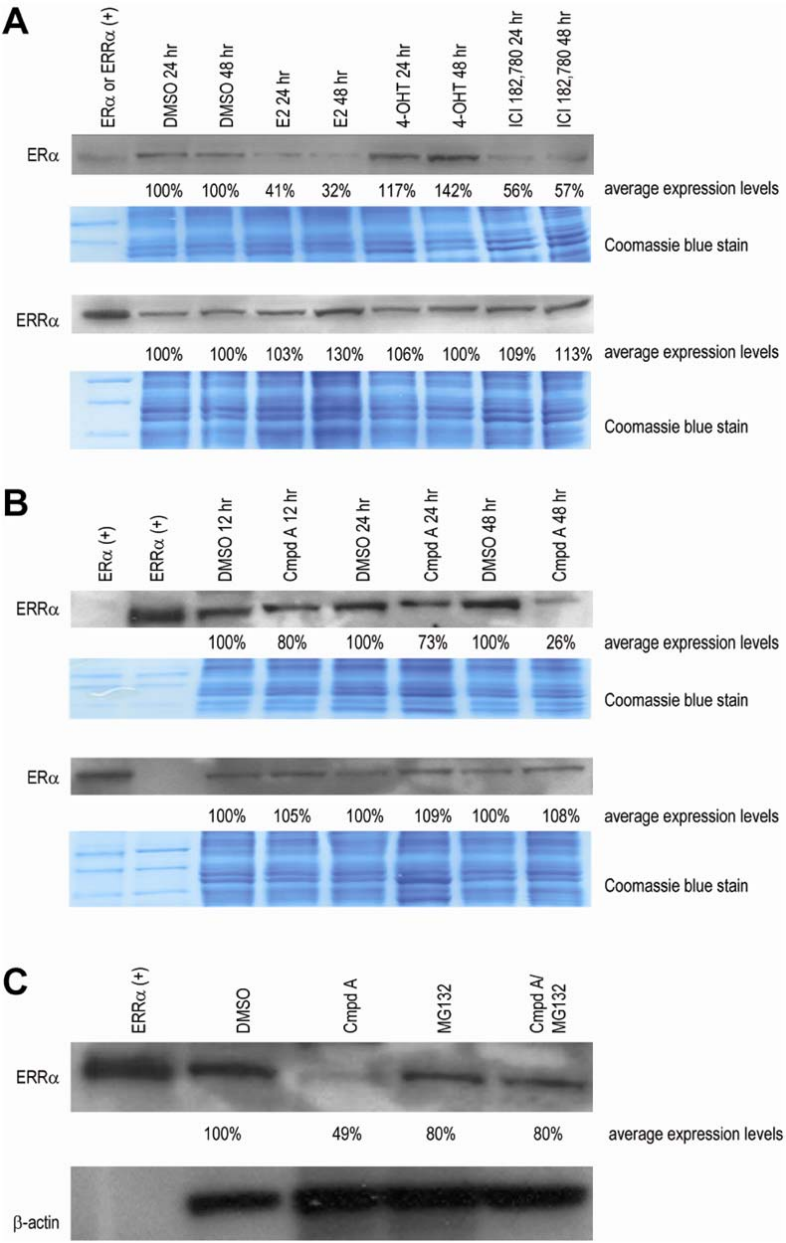
Selective estrogen regulated modulators (SERMs) alter ERα protein stability but have little effect on ERRα. (A) MCF-7 cells were treated with vehicle (DMSO), E2, 4-OHT, and ICI-182,780 for 24 and 48 hours. ERα and ERRα protein levels (nuclear extracts) were analyzed by Western blot. Vehicle does not effect ERα protein stability, E2 and ICI-182,780 lead to degradation, and 4-OHT increases ERα stability (Fig. 4A top). Neither 4-OHT or ICI-182,780 treated cells altered ERRα levels at 24 or 48 hours, while there is a 30% increase in ERRα after 48 hour treatment with E2 (Fig. 4A bottom). (B) MCF-7 cells were treated with vehicle (DMSO) or Compound A for 12, 24, and 48 hours. ERRα and ERα protein levels (nuclear extracts) were analyzed by Western blot. After a 12 hour incubation with Compound A, a 20% reduction of ERRα protein was seen; while after 24 hours a 27% reduction and after 48 hours a 74% reduction of ERRα was exhibited. Additionally, treating cells with ERRα antagonist for 12, 24, or 48 hours yielded negligible ERα protein level changes (Fig. 4B bottom). (C) MCF-7 cells were treated with vehicle (DMSO), Compound A, MG132, or Compound A plus MG132 for 36 hours. ERRα protein levels (whole cell extracts) were analyzed by Western blot. Cells treated with Compound A exhibited a 51% reduction in comparison to vehicle. Addition of MG132 blocks ERRα degradation caused by Compound A. Equal loading of nuclear protein extracts per lane was assessed by Coomassie blue staining of gels (Fig. 4A,B) while additionally; equal loading of whole cell protein extracts per lane was assessed by stripping the nitrocellulose membrane and re-probing with β-actin monoclonal antibody (Fig. 4C). Densitometric quantification of protein levels is described in *Materials and Methods*. All Western blots included human full-length ERα and/or ERRα *in vitro* translated proteins which were used as positive controls. Results shown are representative of three independent experiments.

It has been well established that different ER ligands have different effects on ERα protein stability and degradation. For example, at 48 hours 4-hydroxytamoxifen (4-OHT) or idoxifene increases ERα protein levels, estradiol (E2) or ICI -182,780 induces protein degradation, while others like raloxifene display little effect [34], [35]. Also, given that ERα in some contexts is most likely needed for ERRα activation to occur (Fig. 1), we examined the expression status of ERRα after MCF-7 cells were treated with vehicle (DMSO) or the selective estrogen receptor modulators (SERMs) 10 nM E2, 10 nM 4-OHT, and 10 nM ICI-182,780 for 24 and 48 hours. Vehicle (DMSO) does not effect protein stability, while as previously reported [35] E2 or ICI-182,780 leads to degradation while 4-OHT leads to an increase in ERα (Fig. 4A top). Interestingly, 4-OHT and ICI-182,780 treated MCF-7 cells do not alter ERRα levels, while there is a 30% increase in ERRα after 48 hour treatment with E2 (Fig. 4A bottom). To study the effects of ERRα and ERα stability after treatment with the ERRα ligand Compound A, MCF-7 cells were treated with vehicle (DMSO) or 5 uM Compound A for 12, 24, and 48 hours. Nuclear extracts were isolated and ERRα and ERα protein levels were analyzed by Western blot. After a 12 hour incubation with Compound A, a 20% reduction of ERRα protein was seen (compare lanes 3 and 4, Fig. 4B); while after 24 hours a 27% reduction (compare lanes 5 and 6) and after 48 hours a 74% reduction (compare lanes 7 and 8) of ERRα was detected. Treating cells with the ERRα antagonist for 12, 24, or 48 hours yielded negligible ERα protein level changes (Fig. 4B).

To determine whether down regulation of the ERRα protein is mediated by the ubiquitin proteasome pathway, we treated MCF-7 cells with vehicle (DMSO), 5 uM Compound A, the proteasome inhibitor MG132 (1 uM), or 5 uM Compound A plus 1 uM MG132 for 36 hours. Whole cell extracts were isolated and ERRα protein levels were analyzed by Western blot. Cells treated with the ERRα antagonist (lane 3, Fig. 4C) exhibited a 51% reduction in comparison to vehicle (lane 2). Addition of MG132 slightly reduced ERRα (lane 4 compared to lane 2) while addition of MG132 blocks ERRα degradation caused by Compound A (lane 5 compared to lanes 2, 3, and 4) (Fig. 4C). Thus, our results suggest that Compound A down-regulation of ERRα involves ubiquitin-mediated proteolysis.

### Treatment with the ERRα Antagonist Decreased Association between ERRα and ERRα Targeted Promoters

**Figure 5:**
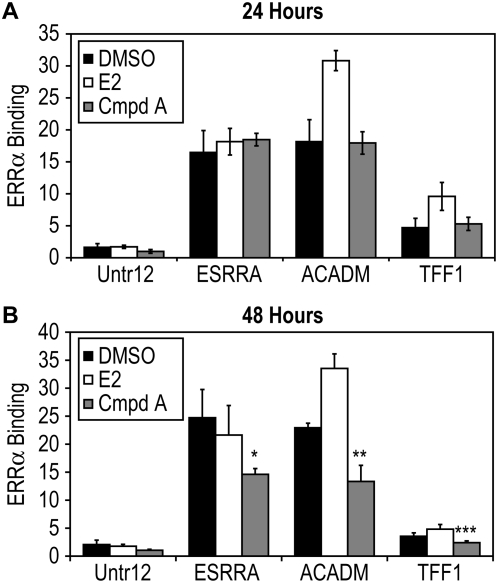
Treatment with Compound A decreases the association of ERRα to ERRα target gene promoters. MCF-7 cells were treated with DMSO, E2, or Compound A for 24 and 48 hours followed by ChIP with anti-ERRα antibody. Quantitative real-time PCR (Q-PCR) was performed with primers targeting estrogen-related receptor response elements (ERREs) in ERRα (*ESRRA*), *ACADM*, and pS2 (*TFF1*). (A) At 24 hours, treatment with Compound A had little or no effect on ERRα binding of target genes ERRα (*ESRRA*), *ACADM*, and pS2 (*TFF1*). (B) At 48 hours significant decreased binding of ERRα (*ESRRA*), *ACADM*, and pS2 (*TFF1*) (P<0.001) was exhibited. All ChIP experiments were independently replicated in triplicate. Differences in binding between vehicle (DMSO) and Cmpd A were measured by ANOVA followed by a student t-test with a 0.05 significance level.

In order to investigate the effect of Compound A on ERRα binding at the promoter region of ERRα target genes (*ESRRA*, *ACADM*, and *TFF1*), chromatin Immunoprecipitation (ChIP) assays were performed after MCF-7 cells were treated with DMSO, 3 pM 17β-estradiol (E2), or 5 uM Compound A for 24 and 48 hours. The cells were fixed, chromatin was immunoprecipitated with anti-ERRα antibody, and quantitative real-time PCR (Q-PCR) was performed with primers targeting well characterized estrogen-related receptor response elements (ERREs) in ERRα (*ESRRA*) [30], [31], *ACADM* [4], [32], and pS2 (*TFF1*) promoters [6], [33]. At 24 hours, treatment with Compound A had little or no effect on ERRα association with these target genes (Fig. 5A), while at 48 hours decreased association of *ESRRA*, *ACADM*, or *TFF1* (*P*<0.001) (Fig. 5B) was demonstrated, suggesting a decreased association between ERRα and ERRα targeted promoters.

### Silencing of ERRα Decreases mRNA levels of ERRα Target Genes but not ERα

**Figure 6:**
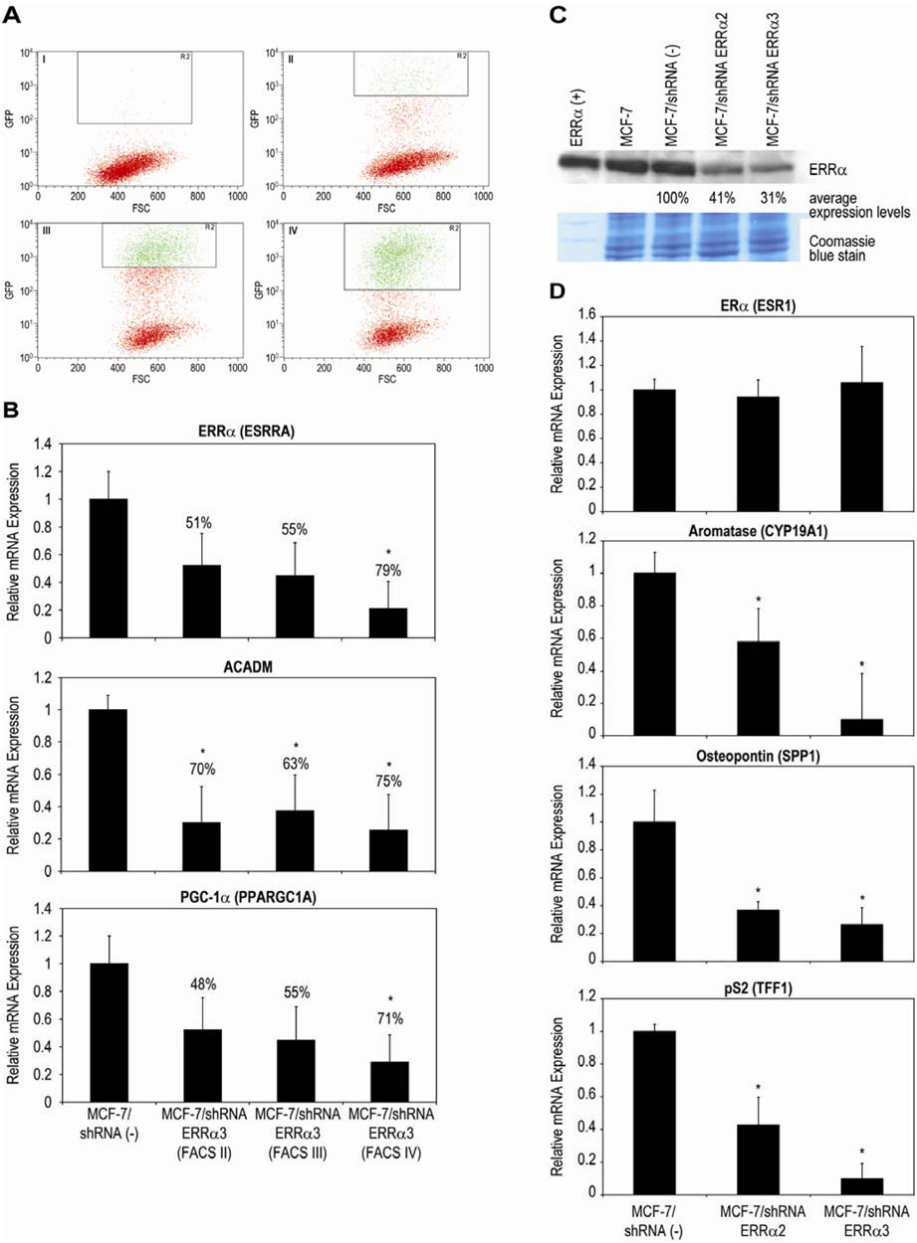
Knock-down of ERRα by shRNA (RNAi) decreases mRNA levels of multiple ERRα target genes. Short hairpin plasmids expressing short hairpin RNA (shRNA) specific for knocking-down ERRα were transfected separately into MCF-7 cells. In addition, a shRNA expressing a scrambled artificial non-specific sequence was transfected. (-) control. (A) MCF-7/shRNA ERRα3 cells underwent four rounds (I–IV) of fluorescent activated cell sorting (FACS) to enrich for GFP expressing cells. FACS is described in *Materials and Methods*. (B) ERRα (*ESRRA*), *ACADM*, and PGC-1α (*PPARGC1A*) mRNA expression was measured by real-time RT-PCR. After 4 rounds of FACS, MCF-7/shRNA ERRα3 cells ERRα (*ESRRA*) mRNA levels were significantly reduced by 79%, *ACADM* levels by 75%, and PGC-1α (*PPARGC1A*) by 71% (*, P<0.05). Differences in relative mRNA expression between MCF-7/shRNA (-) and MCF-7/shRNA ERRα3 were measured by ANOVA followed by a student t-test with a 0.05 significance level. (C) MCF-7, MCF-7/shRNA (-), MCF-7/shRNA ERRα2, and MCF-7/shRNA ERRα3 cells ERRα protein expression was measured by Western blot, equal loading of protein was assessed by Coomassie blue staining of gels, and densitometric quantification are described in *Materials and Methods*. Results shown are representative of three independent experiments. MCF-7/shRNA ERRα2 exhibited 59% less protein verses the negative control, while MCF-7/shRNA ERRα3 cells ERRα protein levels were reduced by 69%. (D) ERα (*ESR1*), aromatase (*CYP19A1*), osteopontin (*SPP1*), and pS2 (*TFF1*) mRNA expression was measured in MCF-7/shRNA (-), MCF-7/shRNA ERRα2, and MCF-7/shRNA ERRα3 cells by real-time RT-PCR after 4 rounds of FACS. Knocking-down ERRα by shRNA (RNAi) led to significant decrease (*, P<0.05) in expression of ERRα target genes aromatase (*CYP19A1*), osteopontin (*SPP1*), and pS2 (*TFF1*) while ERα (*ESR1*) levels were not affected. All real-time RT-PCR results are representative of three independent experiments performed in triplicate.

Does reduction of ERRα expression lead to similar effects seen by antagonizing/down-regulating ERRα with Compound A? Four different (1–4) plasmids (SuperArray Bioscience Corporation) expressing short hairpin RNAs (shRNA) specific for ERRα under the control of the U1 promoter and containing the GFP marker gene were transfected separately into MCF-7 cells. Additionally, an shRNA expressing a scrambled artificial sequence that does not match any human, mouse or rat gene was transfected and used as the negative control. MCF-7/shRNA ERRα3 cells underwent four rounds of fluorescent activated cell sorting (FACS) to enrich for GFP expressing cells (Fig. 6A). Similar results were seen with MCF-7/shRNA ERRα2 (data not shown). ERRα mRNA expression was measured (Fig. 6B-top panel, S2) and as a higher number of GFP expressing cells were sorted and isolated (rounds I–IV), a decrease in ERRα mRNA levels were detected. While only 51% reduction of ERRα (versus the negative control) was measured with cells that underwent two rounds of FACS, after 4 rounds the enriched population exhibited a statistically significant (*P*<0.05) 79% knock-down. Similar results were seen with MCF-7/shRNA ERRα2 (data not shown). Along with ERRα, ACADM and PGC-1α mRNA expression levels were also determined and statistically significant (*P*<0.05) reduction of ACADM and PGC-1α was measured in both after 4 rounds of FACS (Fig. 6B, S2). ERRα protein expression was measured (Fig. 6C) and MCF-7/shRNA ERRα3 cells exhibited 69% less protein versus the negative control, while MCF-7/shRNA ERRα2 ERRα protein levels were reduced by 59%. Similarly to when MCF-7 cells were treated with Compound A (Fig. 2) knocking-down ERRα by shRNA led to significant decreases (*P*<0.05) in expression of ERRα target genes aromatase (*CYP19A1*), osteopontin (*SPP1*), and pS2 (*TFF1*) while ERα (*ESR1*) levels were not affected (Fig. 6D, S3).

## Discussion

Breast cancer therapies continue to be an unmet medical need as an estimated 40,930 (40,480 woman and 450 men) breast cancer deaths are expected in 2008 [36]. There is growing evidence that the orphan nuclear receptor ERRα takes part in breast cancer progression and could be a novel drug target to treat breast cancer [2], [3], [21], [25], [37], [38], [39]. N-[(2Z)-3-(4,5-dihydro-1,3-thiazol-2-yl)-1,3-thiazolidin-2-yl idene]-5H dibenzo[a,d][7]annulen-5-amine, Compound A, is a recently reported ERRα-subtype specific ligand, identified on the basis of disrupting the constitutive interaction between ERRα and nuclear coactivators [21]. In this study, we characterized the molecular mechanism of Compound A in modulating ERRα activity.

First, Compound A inhibits the constitutive transcriptional activity of both endogenous and ectopically expressed ERRα (Fig. 1A). When ERRα was overexpressed in MCF-7 cells, a greater window of repression by Compound A was exhibited, and the overall estrogen responsiveness (measured by an ERE reporter construct) was down-modulated (Fig. 1B) – an interesting event first reported by Kraus and colleagues [26]. Our previous research reports that Compound A specifically binds ERRα [21]. Compound A did not exert a direct effect on ERα either by modulating mRNA expression or altering protein stability. Furthermore, while Compound A does not modulate ERRα mRNA expression, it induces proteasome-dependent ERRα protein degradation (Fig. 2 & 4C). Lanvin and colleagues recently reported the ERRα inverse agonist XCT790 does not act on ERα or ERRα mRNA level, nor does it modify ERα protein stability, but it also induces proteasome dependent ERRα protein degradation [23]. Furthermore, when MCF-7 cells were treated with Compound A for 12 hours and 24 hours, modest 20% and 27% reduction in protein levels are seen (Fig. 4B). But at 48 hours, a robust 74% reduction was observed. When MCF-7 cells were treated with Compound A for 48 hours, followed by ChIP performed with anti-ERRα antibody there was a significant decreased association to the three promoters (*ESRRA*, *ACADM*, and *TFF1*) (Fig. 5B). Therefore, based on our ERRα ChIP (Fig. 5B) and our Western blot data (Fig. 4B, C), the decrease in association of ERRα at the promoter region of ERRα target genes, is most likely due to protein degradation of ERRα caused by Compound A.

It has been previously demonstrated that the ERR antagonist DES interferes with the constitutive interaction between ERRγ and the nuclear coactivator GRIP-1 [13]. Therefore, to investigate whether or not Compound A could antagonize the constitutive interaction between ERRα and nuclear coactivators we performed biotinylated pull-down assays with AIB1, GRIP-1, and PGC-1α nuclear coactivators. Our data suggest that Compound A promotes nuclear coactivator release between the ERRα and AIB1, GRIP-1, or PGC-1α (Fig. 3A,B).

To extend the finding that Compound A is acting like an antagonist and disrupts the constitutive interaction between ERRα and nuclear coactivators, ChIP assays were carried out after MCF-7 cells were treated with vehicle or Compound A. At 24 hours, a significant decrease in the interaction between GRIP-1/and ERRα (DMSO vs. Compound A, *P*<0.001) (Fig. 3C top) was exhibited. In comparison, when MCF-7 cells were treated with the ERRα antagonist for 24 hours, only a 27% reduction in ERRα protein levels were seen (Fig. 4B). Thus the ability of Compound A to directly interfere with cofactor association is demonstrated by ChIP. At 48 hours, decreased binding of ERRα (*ESRRA*), *ACADM*, and pS2 (*TFF1*)/GRIP-1 (DMSO vs. Cmpd A, P<0.001) (Fig. 3C bottom) was demonstrated and at 48 hours, a robust 74% reduction is reported (Fig. 4B). Therefore, at 48 hours, the significant (DMSO vs. Compound A, *P*<0.001) decrease in association between GRIP-1 and ERRα target genes is most likely due to degradation of ERRα protein.

In order to confirm that antagonizing/down-modulating of ERRα by Compound A is specifically acting through ERRα, we used shRNA plasmids to knock-down endogenous ERRα. After four rounds of FACS, excluding ERα (*ESR1*), all other genes were significantly reduced (*P*<0.05) in MCF-7/shRNA ERRα3 cells (Fig. 6B), corroborating the effects seen when MCF-7 cells were treated with the ERRα-subtype specific ligand, Compound A, for 24 hours (Fig. 2). Furthermore, treatment of MCF-7 breast cancer cells with Compound A, leads to inhibition of growth; similarly, MCF-7/shRNA ERRα3 cell growth is also slowed when compared to the control MCF-7/shRNA (-) cell line [39].

It has been well demonstrated that targeting ERα with such drugs as tamoxifen and ICI-182,780 (faslodex) has lead to successful therapy [40], [41]. Therefore, future studies should include treating ERα (+) cells with combinations of tamoxifen, faslodex or an aromatase inhibitor with Compound A. Additionally, it would be of great interest to treat both ERα (-) and tamoxifen resistant cells with Compound A. In the future it will be interesting to study the effects of knocking-down or antagonizing both ERα and ERRα in breast cancer cells. Intriguingly, Lanvin and coauthors recently reported that the XCT790, an ERRα selective inverse agonist, plus the pure anti-estrogen ICI-182,780 potentiates the ICI-182,780 induced ERα degradation inferring XCT790 may enhance the efficacy of ICI-182,780 in breast cancer treatment. Additionally, XCT790+ICI-182,780 dramatically enhanced ERRα degradation verses ERRα degradation induced by just XCT790 [23].

Based on supporting data in the literature an ERRα specific antagonist shows exciting potential as a novel therapy to treat breast cancer. ERRα is expressed in numerous human breast cancer cell lines, breast tumors, and in breast adipose tissue [6], [37], [38]. ERRα expression in human breast carcinomas is significantly associated with an increased risk of disease recurrence or poor clinical outcome [38]. It has been reported that ERRα expression is associated with an adverse, aggressive tumor phenotype correlating with ERBB2 (HER2, NEU) overexpression [37]. Additionally, the ERRα ligand DES slows breast cancer cell growth at high concentrations (*in vitro*) [6] and in the past has been used to treat breast cancer in clinical settings [42]. ERRα antagonists may also be used for ER-negative cancers. BT-20/shRNA ERRα knock-down cell lines were also established and sorted by FACS. Interestingly, after the second round of GFP enrichment, BT-20 (ER-negative) [43] cells carrying shRNA ERRα plasmid, stopped growing (data not shown).

The possibility of an ERRα antagonist to treat breast cancer plus our recent discovery of new ERRα-subtype specific ligands has led to an effort to characterize the mechanisms of action of ERRα specific antagonists. In particular, N-[(2Z)-3-(4,5-dihydro-1,3-thiazol-2-yl)-1,3-thiazolidin-2-yl idene]-5H dibenzo[a,d][7]annulen-5-amine, (termed Compound A) which has the strongest antagonistic effect on the constitutive interaction between ERRα and nuclear coactivators among the ligands we identified [21]. The pure anti-estrogen ICI, 182,780, which is presently being used to successfully treat breast cancer in the clinic [44] has little effect on ERα mRNA levels [35], promotes cofactor disassociation [45], and induces ERα protein proteasome mediated degradation [35], [46]. Similarly, we have shown that Compound A has little effect on ERRα (and ERα) mRNA levels (Fig. 2), promotes cofactor disassociation (Fig. 3), and induces ERRα protein proteasome mediated degradation (Fig. 4). We report Compound A inhibits ERRα transcriptional activity in MCF-7 cells, and ERRα target genes such as pS2 (*TFF1*), osteopontin (*SPP1*), and aromatase (*CYP19A1*) mRNA levels decreased upon treatment with the ERRα ligand. Knocking-down ERRα (by shRNA) led to similar genomic effects seen when MCF-7 cells were treated with our ERRα antagonist; thereby confirming Compound A's target as ERRα. Our studies presented here improve our understanding of the mechanism of action of the ERRα specific antagonist, Compound A.

## Supporting Information

Figure S1 Mean delta CT values that correspond to the relative gene expression displayed in Figure 2. See Materials and Methods (Real-time RT-PCR) for more information on mean delta CT values.
(0.06 MB TIF)

Figure S2 Mean delta CT values that correspond to the relative gene expression displayed in Figure 6B.
(0.01 MB TIF)

Figure S3 Mean delta CT values that correspond to the relative gene expression displayed in Figure 6D.
(0.01 MB TIF)
